# Supplementary material for: Causal Relationship between Aging and Anorexia Nervosa: A White-Matter-Microstructure-Mediated Mendelian Randomization Analysis
Source: Biomedicines. 2024 Aug 16;12(8):1874. doi: 10.3390/biomedicines12081874 (PMC11351342; doi:10.3390/biomedicines12081874)
Supplement: Supplementary file 1 [file biomedicines-12-01874-s001.zip › Supplementary Figure/Figure S4/Singe SNP plot of MR analysis for TL to ubm-b-1808.pdf]

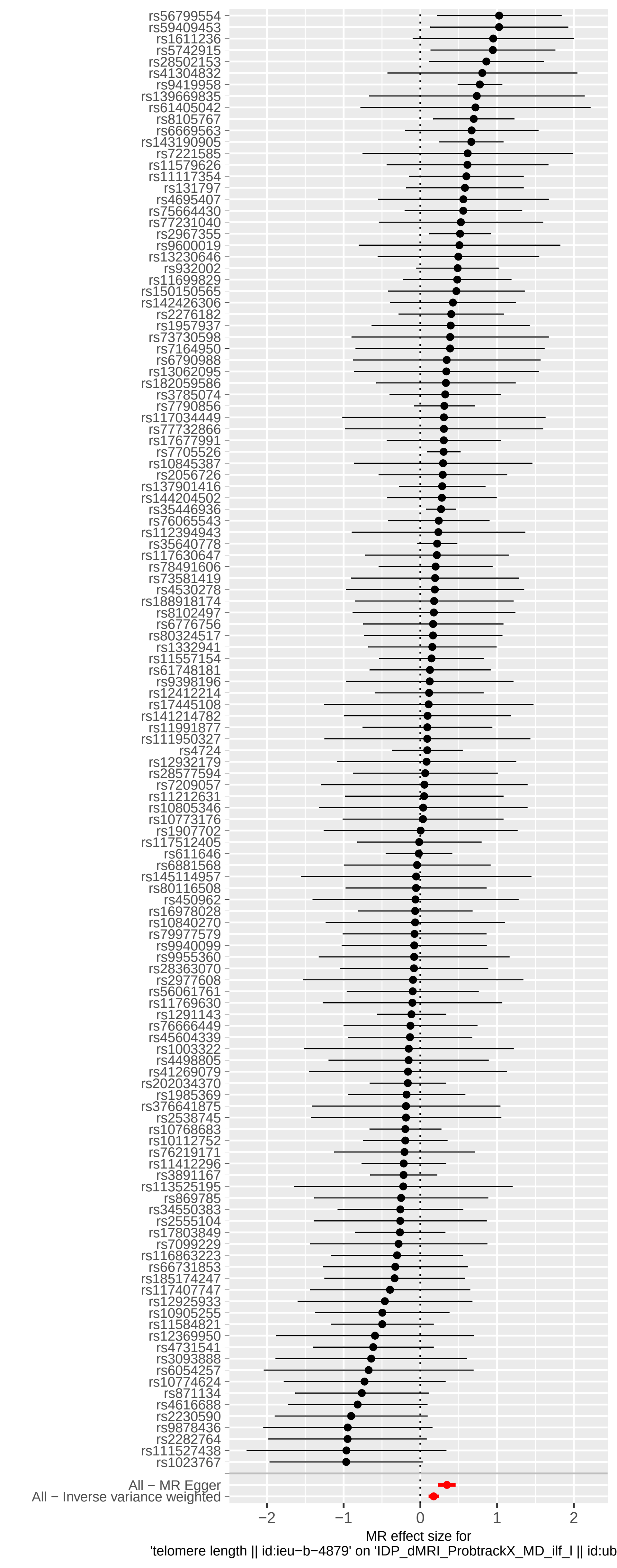
rs56799554
rs59409453
rs1611236
rs5742915
rs28502153
rs41304832
rs9419958
rs139669835
rs61405042
rs8105767
rs6669563
rs143190905
rs7221585
rs11579626
rs11117354
rs131797
rs4695407
rs75664430
rs77231040
rs2967355
rs9600019
rs13230646
rs932002
rs11699829
rs150150565
rs142426306
rs2276182
rs1957937
rs73730598
rs7164950
rs6790988
rs13062095
rs182059586
rs3785074
rs7790856
rs117034449
rs77732866
rs17677991
rs7705526
rs10845387
rs2056726
rs137901416
rs144204502
rs35446936
rs76065543
rs112394943
rs35640778
rs117630647
rs78491606
rs73581419
rs4530278
rs188918174
rs8102497
rs6776756
rs80324517
rs1332941
rs11557154
rs61748181
rs9398196
rs12412214
rs17445108
rs141214782
rs11991877
rs111950327
rs4724
rs12932179
rs28577594
rs7209057
rs11212631
rs10805346
rs10773176
rs1907702
rs117512405
rs611646
rs6881568
rs145114957
rs80116508
rs450962
rs16978028
rs10840270
rs79977579
rs9940099
rs9955360
rs28363070
rs2977608
rs56061761
rs11769630
rs1291143
rs76666449
rs45604339
rs1003322
rs4498805
rs41269079
rs202034370
rs1985369
rs376641875
rs2538745
rs10768683
rs10112752
rs76219171
rs11412296
rs3891167
rs113525195
rs869785
rs34550383
rs2555104
rs17803849
rs7099229
rs116863223
rs66731853
rs185174247
rs117407747
rs12925933
rs10905255
rs11584821
rs12369950
rs4731541
rs3093888
rs6054257
rs10774624
rs871134
rs4616688
rs2230590
rs9878436
rs2282764
rs111527438
rs1023767
All − MR Egger
All − Inverse variance weighted
−2
−1
0
1
2
MR effect size for
'telomere length || id:ieu−b−4879' on 'IDP_dMRI_ProbtrackX_MD_ilf_l || id:ub
